# Meta-Analysis of the Global Mortality Rate Due to Infection in Burn Patients Admitted for Plastic Surgery

**DOI:** 10.7759/cureus.67425

**Published:** 2024-08-21

**Authors:** Shahan Saleem, Ayesha Rehman, Amna Akbar, Amir Iqbal Ali, Sarosh Khan Jadoon, Muhammad Iftikhar Khattak, Adnan Mehraj

**Affiliations:** 1 Cosmetic, Reconstructive and Burn Surgery, Jinnah Burn and Reconstructive Surgery Center, Lahore, PAK; 2 Surgery, Divisional Headquarters Teaching Hospital, Mirpur, PAK; 3 Emergency and Accident, District Headquarter Hospital, Muzaffarabad, PAK; 4 General Surgery, Combined Military Hospitals, Muzaffarabad, PAK; 5 Research and Development, Celestial &amp; Dimanche, Muzaffarabad, PAK; 6 Surgery, Azad Jammu Kashmir Medical College, Muzaffarabad, PAK

**Keywords:** mortality, death, bacterial infection, microbial infection, plastic surgery, burn

## Abstract

Burn patients are generally prone to infection, which causes the patient's condition to be even worse. However, there is no study regarding the difference between the mortality rate of infected and non-infected patients. Therefore, the aim was to identify and compare the global mortality rate between infected and non-infected patients who were admitted to plastic surgery units. We searched PubMed, ScienceDirect, and Google Scholar and finally included five articles for this meta-analysis. We determined the odds ratio (OR) value by forest plot and assessed the study bias by a funnel plot. We also analyzed the quality and heterogeneity. The OR was determined as 0.43 (95%CI: 0.07-2.60), indicating a higher mortality rate in infected burn patients as compared to non-infected patients. The funnel plot showed no significant study bias. The quality of the studies was assessed high as well, and the heterogeneity was determined significant (I^2^>75%). The sensitivity analysis with the fixed effect model reconfirmed our main outcome. However, as a study limitation, we could not specifically determine the impact of strain-specific infection on the mortality rate and could not find more relevant research regarding this issue. We conclude that the overall non-infected burn patient mortality rate is lower as compared to the infected burn patients; however, non-infected patients can be prone to death if the burn degree is higher, the respiratory organ is injured, or the treatment is poor or delayed.

## Introduction and background

Burn injuries are one of the common unexpected incidents that cause morbidity and mortality worldwide. Approximately, 40,000 cases of burn admission are observed yearly in the United States [1]. Burn patients are usually prone to infection as the burnt area has no skin protection, and the patients are weak and immunocompromised. This infection is the primary cause of morbidity, as well as a higher rate of mortality [2-4]. There are different ways by which an individual can be exposed to burn incidents, including flame, scald, electric burn, chemical burn, radiation, and so on [5]. Patients who face severe burns, approximately >30% of the total body surface area (TBSA), remain at high risk of infection. However, burn patients who have <30% TBSA burn are still at risk [6]. Several forms of infection may occur in these patients, including pneumonia, invasive and/or non-invasive wound infection, bloodstream infection (BSI), and urinary tract infection (UTI) [6]. Among the infection-causing microorganisms, bacteria (e.g., Enterococcus spp., methicillin-resistant Staphylococcus aureus (MRSA), vancomycin-resistant Enterococcus (VRE), coagulase-negative Staphylococcus, Pseudomonas aeruginosa, Escherichia coli, Klebsiella pneumoniae, Serratia marcescens, Acinetobacter spp., and Bacteroides spp.), fungi such as molds (i.e., Rhizopus, Mucor, Aspergillus) and yeast (i.e., Candida), and viruses (e.g., Cytomegalovirus, herpes simplex virus, and varicella-zoster virus) are generally found as common and harmful [4,7,8].

Usually, the main challenge of plastic surgery among burn patients is the reconstruction of the burned area, especially with the replacement of damaged skin [3]. Since burns cause rigorous damage to the skin barrier and cause loss of body fluids, the plastic surgeon's primary objective is to remove dead skin and tissue and repair the continuity of tissue and skin in the burn area. Plastic surgery may include autografts, reliable banked tissues, and/or adipose stem cells (ASC) [3,9-11].

After burn and hospital admission within the surgical preparation period before surgery, the risk of infection is high. However, no study determined the chances of mortality of burn patients due to infection. Therefore, in this study, we investigated the differences in mortality rates between infected and non-infected hospitalized burn patients.

## Review

Methodology

Study Guidelines, Search, and Eligibility

The study was conducted admiring the Preferred Reporting Items for Systematic Reviews and Meta-Analyses (PRISMA) guideline following previous studies [12,13]. We searched three different databases, including PubMed, ScienceDirect, and Google Scholar, with specific keywords, such as “plastic surgery”, “burn”, “bacteria”, “infection”, etc. Advanced search was done in PubMed and ScienceDirect using “title and abstract” and “title, abstract, keywords” with Boolean operators. In Google Scholar, the same keywords were used using “allintitle” in the search bar. The final search was done in July 2024. After the search, only the research articles that had data regarding the mortality rate among burn patients and compared the rate with non-infected and infected groups were selected as the eligible articles. Other than that, all the review articles, case reports, correspondence, letters to the editor, and editorials were excluded.

Data Extraction and Quality Assessment

The main data for the meta-analysis extracted were the event and total number of burn patients of the infected and non-infected groups, from each included study. Additionally, to evaluate the study characteristics, data of study type, study location, participants' demographics including total number of study participants, male and female participant numbers with percentage, mean age, study settings, reason for burn, and the most frequent reason for infection were extracted from each study. The quality of the selected studies was checked by answering the quality measuring questions from the National Institutes of Health (NIH) and the University of North Carolina (UNC) [14,15]. If the answer to the question was found, the study got 1 point for yes (Y), and if not found, then 0 points for no (N). Ultimately, for nine different questions, the total obtained points were converted into percentages to estimate the total score and thus evaluate the quality of the selected studies following previous articles with slight modifications [16]. The percentage score >70% was regarded as high quality, and <50% was regarded as low quality. The score in between was regarded as moderate quality.

Study Biases and Meta- and Sensitivity Analyses

Study bias analysis was done to observe the asymmetry of the included studies using a funnel plot following previous studies. The heterogeneity, which indicates the variation in the study outcomes among different studies, was determined by I2 value, where I^2^>75% was regarded as the significant heterogeneity [16,17]. The meta-analysis with the main extracted data of the included studies was also done using a forest plot with a random effect model. The main target was identifying the odds ratio (OR) and the 95% confidence interval (95% CI) through the forest plot. Another forest plot was created using a fixed effect model to observe the reproducibility and sensitivity of the main forest plot [18]. All these analyses were done using RevMan software (version 5.4; Cochrane Collaboration, London, UK).

Result

Study Inclusion and Characteristic

**Figure 1:**
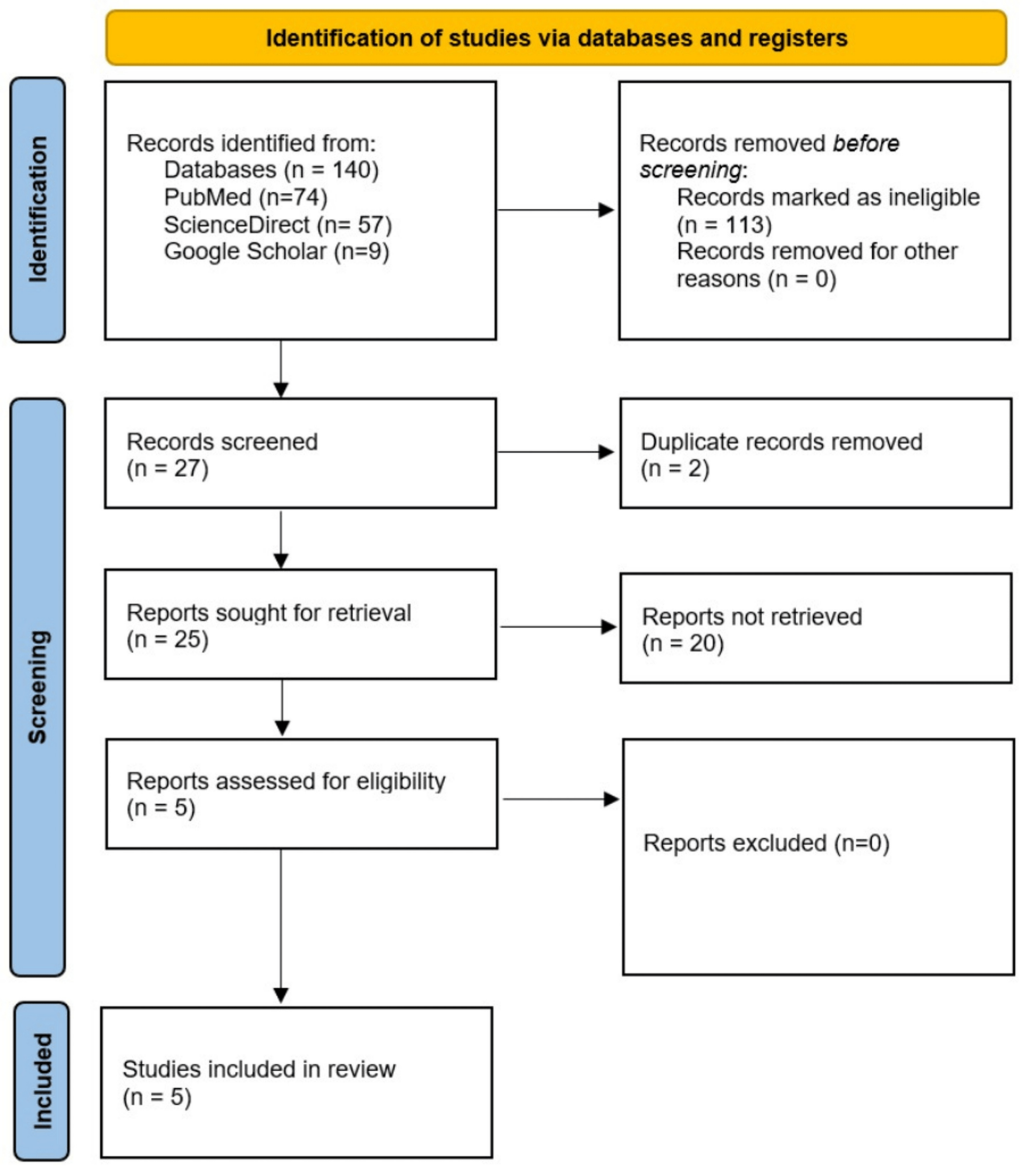
The PRISMA diagram of the study inclusion strategy. PRISMA: Preferred Reporting Items for Systematic Reviews and Meta-Analyses

We initially found 140 articles after applying the search strategy in PubMed (n=74), ScienceDirect (n=57), and Google Scholar (n=9). A total of 113 articles were primarily excluded due to the ineligibility of not being full-length research articles. Of the remaining 27 articles, two were excluded due to study duplication, and 20 were excluded due to the topic's irrelevance. Finally, five articles were selected and included in this study. The sequential search and study inclusion and exclusion process using the PRISMA flow chart is presented in Figure 1.

The study characteristics were carefully assessed and extracted from the five included studies [19-23]. The detailed characteristics of the included studies are presented in detail in Table 1.

**Table 1:** Investigation of the study characteristics. NR: Not reported

Study ID	Study type	Location	Participants demographics				Study settings	Reason of burn	The most frequent reason for infection
			Total (n)	Male (n, %)	Female (n, %)	Age (mean/mean ± SD)			
Belba et al., 2013 [19]	Clinical cohort	Albania	181	113 (62.5)	68 (37.6)	19.9 ± 22.1	ICU, University Hospital Centre (UHC), Tirana	Scald, flame, chemical, electric	Pseudomonas aeruginosa
Ruiz-Castilla et al., 2020 [20]	Single-center cohort	Spain	24	22 (91.7)	2 (8.3)	54	Burns Unit of the Plastic and Reconstructive Surgery Department Vall d’Hebron University Hospital, Barcelona	NR	Pneumonia
Bang et al., 1999 [21]	Clinical cohort	Kuwait	1213	812 (66.9)	401 (33.1)	23	Al-Babtain centre for plastic surgery and burns	Flame, Scald, electric	Streptococcus
Amissah et al., 2017 [22]	Cohort	Ghana	62	37 (60)	25 (40)	25	Reconstructive Plastic Surgery and Burn Center of KBTH	Flame, gas, scald, chemical, electric, acid	Staphylococcus aureus
El Hamzaoui et al., 2020 [23]	Cohort	Morocco	126	64 (50.80)	62 (49.20)	22.50 ± 18.84	Service of Burns and Plastic Surgery ward located at Mohammed V Hospital (378 beds), Meknes city	Flame, hot water, electricity, hot tea	Staphylococcus aureus

Quality and Heterogeneity Assessment

The quality of all the selected studies was high, with four studies obtaining 88.8% and one study obtaining a 100% score (Table 2).

**Table 2:** Assessment of the study quality. Here, Q1. Was the research question appropriate? Q2. Is the target/study population clearly defined? Q3. Were any inclusion and/or exclusion criteria mentioned? Q4. Was any time frame mentioned? Q5. Are non-responders clearly described? Q6. does the sample represent the target population? Q7. Were data collection methods standardized? Q8. Was the measuring kit/tool validated? Q9. Did the authors use statistical analyses? Y=Yes (1 point), N=No (0 point)

Study ID	Q1	Q2	Q3	Q4	Q5	Q6	Q7	Q8	Q9	Total score (%)
Belba et al., 2013 [19]	Y	Y	Y	Y	N	Y	Y	Y	Y	88.8
Ruiz-Castilla et al., 2020 [20]	Y	Y	Y	Y	N	Y	Y	Y	Y	88.8
Bang et al., 1999 [21]	Y	Y	Y	Y	N	Y	Y	Y	Y	88.8
Amissah et al., 2017 [22]	Y	Y	Y	Y	Y	Y	Y	Y	Y	100
El Hamzaoui et al., 2020 [23]	Y	Y	Y	Y	N	Y	Y	Y	Y	88.8

**Figure 2:**
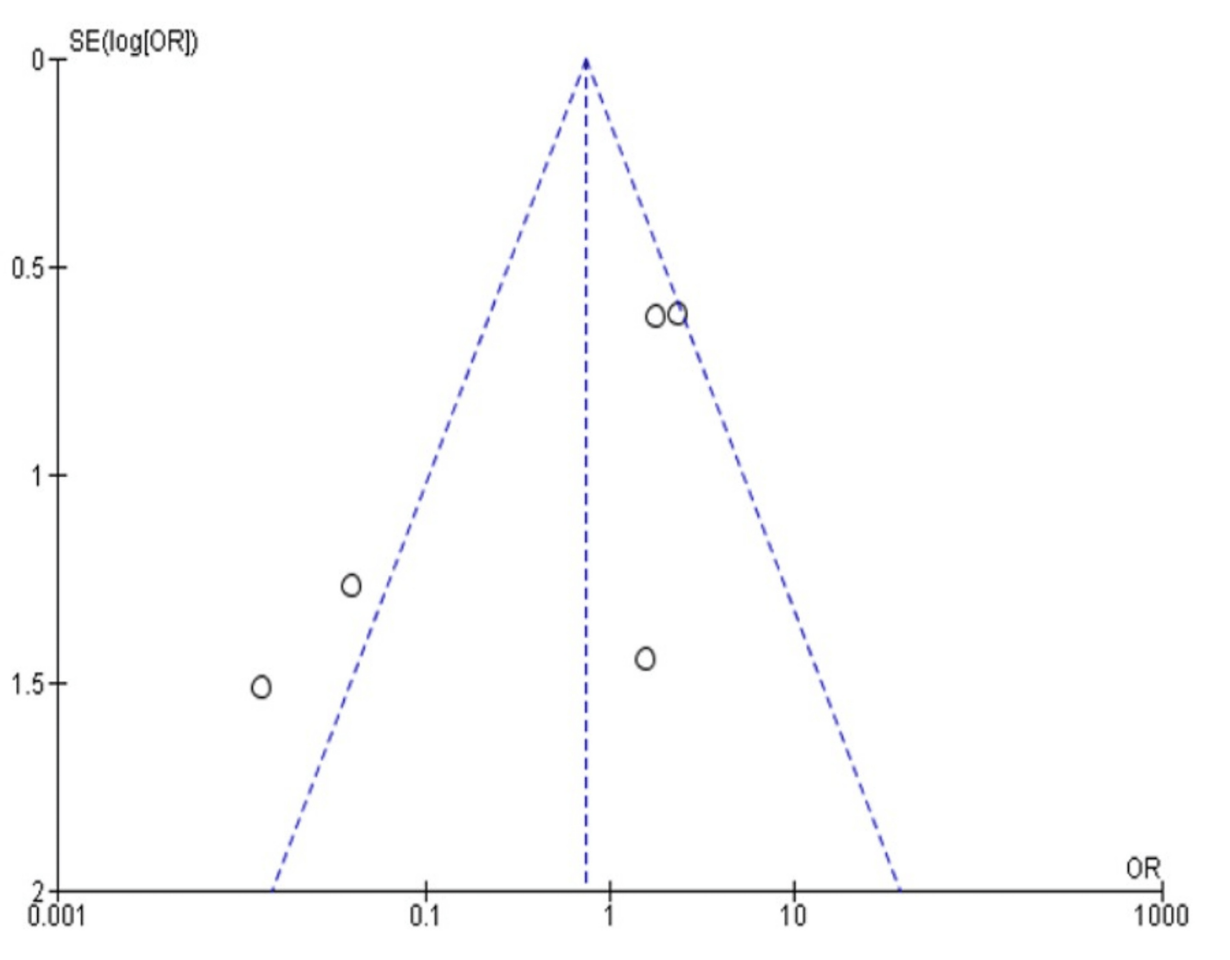
Funnel plot finding the possible source of heterogeneity through the asymmetry assessment. Here, no significant outliers or asymmetry was noticed. Therefore, all the studies were included for further meta-analysis.

Additionally, the study bias assessment through the funnel plot found no significant study asymmetry or outliers (Figure 2).

This reconfirmed that all our included studies were symmetrical, without any major asymmetry for the meta-analysis. Additionally, the heterogeneity was found to be significant (I^2^=78%). 

Meta-Analysis and Sensitivity

**Figure 3:**
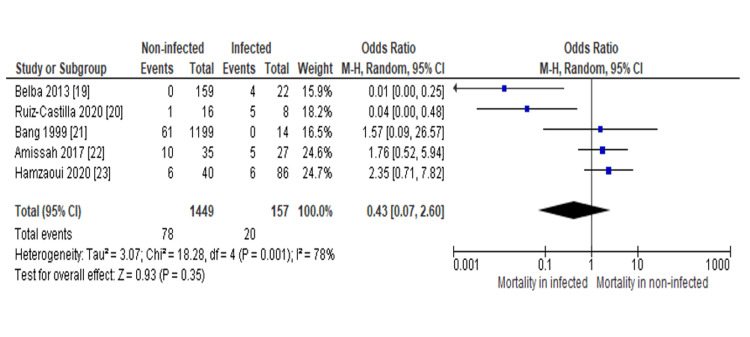
Forest plot assessing the odds ratio (OR) of the mortality rate between infected and non-infected burn patients. The overall OR was identified using Mantel–Haenszel statistics and the random effect model, and the value was found 0.43, with a 95% confidence interval (95%CI) of 0.07 to 2.60. Additionally, the weight and OR of each study have been expressed as well. Heterogeneity was found to be I^2^=78%, which can be referred to as significant. Source: Refs [19-23]

The main forest plot using the random effect model found an OR of 0.43 (95%CI: 0.07-2.60), which supported that the mortality rate was higher in infected burn patients as compared to non-infected burn patients (Figure 3).

**Figure 4:**
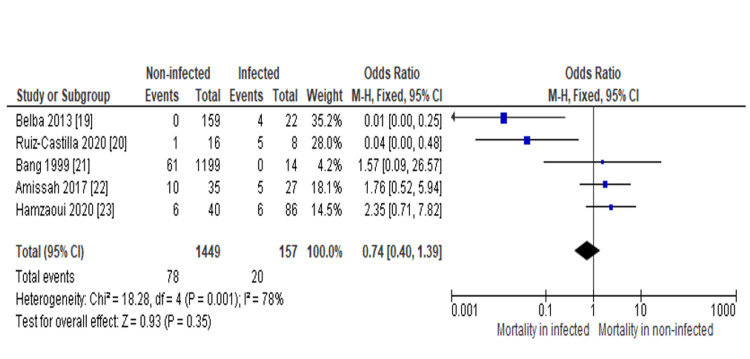
Fixed effect model of the forest plot showing a slightly enhanced odd ratio as compared to the main outcome. The overall OR was identified using Mantel–Haenszel statistics and the fixed effect model, and the value was found 0.74 with a 95% confidence interval (95%CI) of 0.40-1.39. Additionally, the weight and OR of each study have been expressed as well. Source: Refs [19-23]

However, to reinvestigate the sensitivity and reproducibility of the main meta-outcome, the fixed effect model was used to reanalyze the same plot. As a result, OD was determined to be 0.74 (95%CI: 0.40-1.39), confirming our main analysis to be accurate (Figure 4).

Discussion

Burn injuries are dangerous and require immediate treatment to reduce the discomfort, pain, morbidity, and mortality rate, as well as to minimize exposure to microorganisms. However, based on the degree of burn, the treatment should be given. There are three degrees of burn, that is, first, second, and third degrees, based on the depth of the burn [2,24]. However, all of them are enough to be infected by microorganisms if exposed to an unsafe environment for a long period [2]. Additionally, we found that flame is the most common cause of burn, followed by scald, chemical, electric, and hot water burns. Again, in most cases, male burn patients were greater in number as compared to female patients (Table 1).

In this study, we investigated the mortality rate among the infected and non-infected burn patients, where we found that infected burn patients had a higher mortality rate than non-infected patients with an OR of 0.43 (95%CI: 0.07-2.60) (Figure 3). This OR value and the CI range indicate a moderately strong correlation between a higher mortality rate and infection. However, analyzing the studies individually, we observed that three of the studies out of five had a slightly higher mortality rate in non-infected burn patients than in infected. Among them, El Hamzaoui et al. [23] had the highest OR (2.35 (95%CI: 0.71-7.82)), followed by Amissah et al. [22] (1.76 (95%CI: 0.52-5.94)) and Bang et al. [21] (1.57 (95%CI: 0.09-26.57)). These OR (>1) values and CI ranges regarding these studies indicated that non-infected burn patients also face fatality, which in these cases were higher as compared to the infected burn patients. These findings indicate that there are chances of mortality for any burn patients.

Usually, infections in burn patients make the situation worse and can lead to fatality. The reason behind this is that infection may create bloodstream infection, and sepsis, and even can invite other dangerous pathogens to make the situation of the patients even more vulnerable as the burn patients already face weak immunity [19]. Although we found death cases of non-infected burn patients were higher than the infected burn patients in a couple of studies, there were several reasons behind it. According to El Hamzaoui et al. [23], the difference in the mortality rate between infected and non-infected was not statistically significant. They also claimed that the higher rate of non-infected mortality was due to the respiratory site burn, and the degree of burn was high [23]. Amissah et al. [22] indicated that diagnostic availability, immediate approach, and proper treatment for infected burn patients minimized the mortality rate. Nevertheless, they stated that the lack of screening of other bacterial strains that are usually involved in nosocomial infection may be a limitation of their unusual finding regarding the higher mortality in non-infected burn patients [22]. In the case of Bang et al. [21], most of the infected cases had less burn percentage, and in major cases, the source microorganism was determined in the throat rather than the burn wound. Therefore, combined treatment with clinical care and antibiotic therapy was effective enough for them to survive even after being infected. Additionally, the mortality rate in non-infected burn patients was high, which may be due to the enhanced burn degree (>60%), as well as the age factor where around 29.6% were below five years and 1.9% were more than 60 years of age among 1213 burn patients [21].

Different types of microorganisms can cause infection in burn wounds [4]. However, we determined Staphylococcus aureus to be the most prevalent burn wound microorganism, followed by Streptococcus, pneumonia-causing microorganisms, and Pseudomonas aeruginosa (Table 1). As the wounds of burn patients are already exposed to the environment, aseptic conditions should be maintained during the treatment to avoid the attack and progression of microorganisms and infection. During treatment hydrotherapy devices, surgery, and general equipment, treatment room, hands, and clothing of doctors must be ensured to be sterile and decontaminated. Additionally, the chances of nosocomial infection should strictly deteriorate during the treatment and recovery period [2,4]. 

To prevent infection in burn patients, early screening of microorganisms, identification of the specific strain that may cause infection, monitoring of the efficacy of the ongoing treatment, and empiric and perioperative antibiotics and medicines, identifying the chances of cross-colonization and ultimately preventing the unexpected nosocomial infection or transmission of microorganisms is crucial [2,6]. The current findings of this study indicate the plausible risk of mortality due to infection in burn patients. Therefore, the infection risk needs to be mitigated among hospitalized burn patients before and after the surgery period to reduce the mortality rate. Surgery devices, equipment, room and everything needs to be sterile to reduce the infection. Nosocomial infection also needs to be monitored throughout the surgery period. Nevertheless, more comprehensive research articles are required regarding the specific bacteria-based infection and its impact on the mortality rate so that we can have a clearer scenario that would enable hospitals and physicians to take early and more effective treatments, including specific antibiotic doses and necessary steps to prevent and control the rate of infection thus the rate of mortality.

Limitations

This study could only analyze the overall effect of infection on burn patient mortality and could not specifically determine the strain-based impact of the mortality rate. We could not search more databases due to the lack of full accessibility in those databases. We could not find more eligible studies to be added to this meta-analysis; therefore, further research is required focusing on this subject.

## Conclusions

Infection in burn patients can make the situation even worse, raising the chances of mortality. Although our findings suggest higher mortality rates in infected burn patients who came to plastic surgery hospital units, we have also found that mortality rates can be high even in high-degree non-infected burn patients. Therefore, before general or plastic surgery, patients must be taken care of deliberately maintaining the sterile conditions and environmental hygiene to avoid plausible infection and minimize the mortality rate.

## Appendices

**Table 3:** Search strategy in different databases

Databases	Search strategy	The number of articles retrieved	The overall number of articles retrieved
PubMed	((plastic surgery[Title/Abstract]) AND (burn[Title/Abstract])) AND ((((bacteria[Title/Abstract]) OR (infection[Title/Abstract])) OR (antibiotic[Title/Abstract])) OR (bacterial infection[Title/Abstract]))	74	74
ScienceDirect	Title, abstract, keywords: plastic surgery burn bacterial infection	4	57
	Title, abstract, keywords: plastic surgery burn infection	34	
	Title, abstract, keywords: plastic surgery burn bacteria	5	
	Title, abstract, keywords: plastic surgery burn antibiotic	14	
Google Scholar	allintitle: plastic surgery burn bacteria	3	9
	allintitle: plastic surgery burn infection	3	
	allintitle: plastic surgery burn antibiotic	3	
	allintitle: plastic surgery burn bacterial infection	0	
